# Biology to the Fore Again

**Published:** 1915-12

**Authors:** 


### Biology to the Fore Again.

In a recent address before the Scientific Society of San Antonio, Professor Julian S. Huxley of the Rice Institute, brought forth some very pertinent arguments to show that specialization on the part of the State caused degeneration of the individual. In his lecture, "Biology, the Individual and the State," he drew upon the facts in biology to make this analogy in the case of the State. For example, the ant family, he pointed out, was highly specialized, each individual in the colony, male, female, and neuter sexes performed their special functions for the good of the whole, and yet as individuals were useless. Likewise, the jellyfish, the hydra and other vegetable and animal forms in exercising a specialized function accomplished nothing for themselves or for the advancement of their species. So, with the individual in society, when confined too narrowly to a specialized work, it does not permit of a broad and useful life such as will assure the greatest enjoyment to himself and usefulness to the State.

As a further example of specialization, the speaker cited the effect of the war in Europe. Each country is specializing in warfare. As a result, other callings and interests are being destroyed. The best manhood and the strongest intellectuals are sacrificed on the battlefield in the country's vain effort to accomplish just the one thing.

He further pointed out how the State must safeguard against this tendency by providing shorter hours for labor and better opportunities for the individual to acquire more general knowledge.

We do not contend against any of his arguments, though it might be pointed out that, while the individual himself might be limited in his enjoyment of a larger life by following some specialized line, he does perform a genuine service to the State, when his labor is taken as a part of the whole. We can see the abuse and dangers this sort of specialization will lead into when it attempts to assume all the responsibility. For example, in the medical profession, specialism works harmfully, in many cases, when not safeguarded by the conservative influence of a general knowledge of the whole profession.

Again, we are not so sure that the analogy is quite correct between the lower animal and man. In the former the action is purely mechanical or instinctive, whereas with man his actions are influenced by his psychical and reasoning faculties. This position we believe would hold good, unless the professor argues that the individual by specializing becomes more mechanical and thus loses his psychic element.

Whatever conclusion might be reached, the point we wish to emphasize mainly is that more and more the principles of biology are relied upon to point out the fundamentals of government and the principles of philosophy.

